# Exploring Ambulatory Continuous Inotropes as a Bridge to Recovery in Advanced Heart Failure Secondary to Amphetamine-Induced Cardiomyopathy

**DOI:** 10.1016/j.cjco.2024.10.013

**Published:** 2024-11-16

**Authors:** Asma Babar, Bastien Vallée-Marcotte, Anton Savin, Mathieu Bernier, Kim O’Connor, Mario Sénéchal

**Affiliations:** a Department of Cardiology, Québec Heart and Lung Institute, Québec City, Québec, Canada; b Department of Medicine, McGill University, Montréal, Québec, Canada

**The increasing prevalence of amphetamine use has become a major public health concern in Canada. Among the many cardiovascular impacts of amphetamine use, dilated cardiomyopathy is emerging as a rare but morbid complication in young adults. This case report highlights the evolution of a patient with amphetamine-induced cardiomyopathy. We present a common clinical presentation of this disease and a novel approach to address certain treatment challenges in advanced heart failure in this patient population.**

## Case

### History of presentation

A 32-year-old man with a decade-long history of amphetamine misuse presented to the emergency department with a 3-month history of progressive exertional dyspnea, orthopnea, and anasarca. No chest pain, palpitations, or viral symptoms were reported. He was tachycardic (120 bpm), had low blood pressure (80/50 mmHg), and had normal oxygen saturation (95% in ambient air). Cardiac auscultation identified an S3 and a holosystolic murmur in the mitral area. Pulmonary congestion signs, elevated jugular venous pressure, positive hepatojugular reflex, bilateral leg edema, and cool extremities were noted.

### Past medical history

The patient consumed 5-8 amphetamine tablets daily, smoked tobacco, and consumed alcohol occasionally. He reported no personal or familial history of heart disease or sudden cardiac death. He did not take any medication or natural health product.

### Investigations

The initial electrocardiogram revealed sinus tachycardia with nonspecific repolarisation changes in the lateral leads. Chest x-ray showed an increased cardiothoracic index at 0.6 with signs of vascular cephalization and pulmonary congestion. Comprehensive laboratory analysis revealed kidney injury (creatinine: 168 μmol/L, normal [N]: 50-110 μmol/L), hyponatremia (sodium: 124 mmol/L, N 135-145 mmol/L), and metabolic acidosis (pH 7.37, partial pressure of carbon dioxide 28 mmHg, and HCO_3_^−^ 16 mmol/L), with elevated serum lactate (4.4 mmol/L, N <1.6 mmol/L). He had overt hepatic dysfunction (alanine aminotransferase: 7584 U/L, N <45 U/L; alanine transaminase: 4402 U/L, N <60 U/L; gamma-glutamyltransferase: 170 U/L, N <75 U/L; bilirubin: 25 μmol/L, N <17 μmol/L; international normalized ratio: 2.5, N <1.2). He had an elevated N-terminal pro–B-type natriuretic protein level (7347 ng/L). There was no significant elevation of troponin levels (troponin I: 16 ng/L, N <79 ng/L) nor inflammatory markers (C-reactive protein: 8.7 mg/L, N <10 mg/L). The patient was not anemic (hemoglobin: 157 g/L, N 140-180 g/L). Computed tomography angiography was performed, which excluded pulmonary thromboembolism and confirmed interlobular septal thickening compatible with pulmonary edema.

Echocardiography showed dilated cardiomyopathy with severe biatrial and biventricular enlargement (left ventricular [LV] end-diastolic volume: 115 mL/m^2^; LV end-diastolic diameter: 70 mm) (Video 1 , view video online). There was also severe systolic LV dysfunction as measured by Simpson’s method, with an LV ejection fraction of 10% with global severe hypokinesis and severe functional mitral and tricuspid regurgitation. Severe right ventricular dysfunction was also present (tricuspid annular plane systolic excursion: 12 mm, S-wave velocity: 7.6 cm/s). A large 4.7 × 1.2 cm thrombus was visualised in the right ventricle.

Considering these findings, the patient was diagnosed with dilated cardiomyopathy and stage C cardiogenic shock, based on Society for Cardiovascular Angiography & Interventions classification. Inotropic support with milrinone (0.250 μg/kg per min) was initiated, and a Swan-Ganz catheter was inserted to facilitate the titration of therapy. Initial hemodynamics were as follows: cardiac index 1.95 L/min per m^2^, pulmonary artery pressures 46/24 mmHg, mean pulmonary artery pressure 35 mmHg, central venous pressure 13 mmHg, pulmonary capillary wedge pressure 24 mmHg, systemic vascular resistance 1812 dynes/cm^5^, and pulmonary vascular resistance 249 dynes/cm^5^. Coronary angiography showed an absence of significant coronary atherosclerosis. Cardiac magnetic resonance imaging confirmed the presence of biventricular dysfunction with left and right ejection fractions of 16% and 18%, respectively. Thrombi were visualised in the right ventricle and no significant area of late gadolinium enhancement was noted. The patient underwent a positron emission tomography scan to exclude cardiac sarcoidosis. Serum protein electrophoresis did not show a monoclonal protein. Of note, we did not perform endomyocardial biopsy.

### Management (medical interventions)

The patient’s clinical course was marked by inotrope dependence, necessitating an approximate 3-month hospitalization. Attempts to wean the patient off inotropic support and optimally escalate goal-directed medical therapy (GDMT) were unsuccessful. The advanced heart failure team was consulted during the hospitalization and the patient was not considered a candidate for advanced therapies, primarily due to recent severe substance abuse and limited potential compliance. Furthermore, he was not eligible for an LV assist device because of severe right ventricular dysfunction. The decision to pursue ambulatory inotropic therapy was taken as a bridge to recovery or decision. A simple chamber implantable cardioverter defibrillator (ICD) and permanent central catheter were installed, and the patient was discharged from the hospital with continuous infusion of milrinone.

The patient was closely followed up at our heart failure clinic with uptitration of his GDMT with doses of sacubitril-valsartan 97+103 mg twice daily, spironolactone 25 mg/d, bisoprolol 5 mg/d, and ivabradine 2.5 mg twice daily. He remained abstinent from amphetamine use, which was confirmed with serial urine toxicology tests. Clinical course was marked by a shock from his ICD for ventricular fibrillation approximately 1 month after hospital discharge. Interrogation of his ICD showed that the episode of ventricular tachycardia was initiated by a premature ventricular contraction with long coupling. The option to hospitalize and reevaluate his eligibility for advanced heart failure therapies was declined by the patient at that time. Amiodarone was subsequently introduced to the patient’s pharmacopeia and his infusion of milrinone was reduced to 0.125 μg/kg per minute.

Serial echocardiography showed an increase of the LV ejection fraction reaching 40% at 6 months after discharge. Progressive clinical improvement was noted, and the patient returned to work at a local hospital on a part-time basis. In light of the favourable clinical trajectory, a decision was made to readmit the patient and undertake a systematic reduction of inotropic support by decreasing milrinone infusion by 0.0625 μg/kg per min every 48 hours. Strict clinical and laboratory evaluation was maintained to ensure that the weaning was well tolerated. After weaning, echocardiography showed stability in LV function with LV ejection fraction at 40% and, notably, the LV end-diastolic diameter was significantly ameliorated at 58 mm (Video 2 , view video online). Hemodynamic values obtained after complete weaning of milrinone are shown in Table 1. The patient has remained in a stable and vital condition at the 1-year mark after cessation of inotropic support, concurrent with ongoing abstinence from amphetamine use.

**Table 1:** Invasive hemodynamic measurements obtained using a pulmonary arterial catheter

	Initial hemodynamics (August 2021)	Hemodynamics at milrinone weaning (June 2022)
Cardiac index (L/min per m^2^)	1.95	2.93
Pulmonary artery pressures (mmHg)	46/24 (35)	23/6 (12)
Central venous pressure (mmHg)	13	0
Pulmonary capillary wedge pressure (mmHg)	24	7
Systemic vascular resistance (dynes/cm^5^)	1812	1096
Pulmonary vascular resistance (dynes/cm^5^)	249	67

## Discussion

### Association with current guidelines/position papers/current practice

Since it was first reported in North America in the late 1980s, amphetamine-associated cardiomyopathy is increasingly recognized as a lethal complication of amphetamine use. Animal and human studies have explored the cardiotoxic effects of amphetamines and proposed pathophysiologic mechanisms, including direct cellular toxicity and chronic overactivation of the sympathetic nervous system.^1^ Other molecular factors have also been reported, including mitochondrial dysfunction, local immune reactions, vasospasm, impairment of nitric oxide release, and local ischemia.^1^ Endomyocardial biopsy studies in patients with amphetamine-related cardiomyopathy have documented a higher degree of inflammation with presence of T lymphocytes and macrophages when compared with age-, sex-, and LV ejection fraction–matched controls with dilated cardiomyopathy.^2^ Prior investigations have shown that this population has a high prevalence of intracardiac thrombi, as was noted in this case report.^3^ In a recent study, about 20% of the patients with toxic cardiomyopathy presented in cardiogenic shock.^4^

Complete abstinence from drug use is the cornerstone of medical management in amphetamine-related cardiomyopathy, in addition to GDMT for heart failure with reduced ejection fraction.^1^ In our clinical experience with patients who have heart failure with reduced ejection fraction due to toxic cardiomyopathy, complete abstinence was associated with LV function recovery of >40% at 21 months in 61% of patients.^4^ Predictors of reversibility are currently in being investigated and data from case reports suggest that the degree of macroscopic fibrosis on cardiac magnetic resonance imaging can be an important prognostic marker.^3^

Although abstinence may be associated with reversibility of cardiac dysfunction, this may be observed after >12 months. When patients present to hospital with overt cardiogenic shock and are unable to be weaned from inotropic support, they do not have the time to wait for amelioration of cardiac dysfunction. In this type of situation, advanced heart failure therapies, such as heart transplant and LV assist device, may be limited because of concerns of potential nonadherence to therapy.^1^ As shown in our case report, the use of continuous intravenous inotropic agents is a viable option to support patients and obtain reverse cardiac remodelling. This approach diverges slightly from guideline recommendations^5^; however, as demonstrated in the case presented, it provides a potential alternative to young patients who would otherwise face an extended hospitalization period.

Ambulatory inotropic therapy is recommended for patients with advanced heart failure refractory to GDMT and device therapy, who are eligible and awaiting mechanical circulatory support or cardiac transplantation. It is also suggested that clinicians consider this therapy for patients as a palliative measure.^5^ In the modern era, evidence supporting the use of ambulatory inotropic therapy is limited to mostly retrospective cohort studies with variable patient populations. In their systematic review and meta-analysis by of 66 studies, including 13 randomized controlled trials (434 patients), Nizamic et al. suggested that ambulatory inotrope infusions can improve New York Heart Association functional class but do not have a significant impact on survival. Of interest, the most common inotrope used was dobutamine (74% of studies) and intermittent infusions were used more than continuous infusions (50.0% vs 32%, respectively).^6^ Unlike intermittent dosing, continuous infusions deliver a steady plasma level of the drug with a more consistent therapeutic effect to ensure a more stable hemodynamic improvement. Data from a recent retrospective analysis from a large US registry by Mody et al., including 1149 patients on continuous outpatient intravenous inotrope therapy, suggest that, in the current era of GMDT, milrinone use was associated with improved survival compared with dobutamine.^7^

At the Québec Lung and Heart Institute, we have created a small home inotropic therapy program that uses intermittent (twice weekly) or continuous milrinone infusion. Currently, 5 patients (mean age: 54 ± 15 years; etiology: 60% ischemic cardiomyopathy; months on perfusion: 42 ± 45 months), all with advanced heart failure and considered ineligible for a transplant or ventricular assistance device, benefit from continuous milrinone infusions. Astonishingly, 1 of our patients has received a continuous milrinone infusion for the past 10 years. Ten other patients are on intermittent milrinone perfusion. Our protocol for the use of ambulatory milrinone infusion and pictures of the elastomeric infuser are presented in Supplemental Appendix S1 (ambulatory milrinone protocol infusion) and Supplemental Figure S1 (elastomeric infuser) and S2 (peripherally inserted central catheter line) files. Clearly, a randomized controlled trial in this growing patient population is warranted.

Many of the studies conducted on the use of ambulatory inotropes were from the pre-ICD era. Our patient’s episode of ventricular tachycardia, which was treated with appropriate ICD shock, confirms the importance of ICD implantation in those receiving home inotropic support as a bridge to recovery.

## Conclusions

The medical management of patients with amphetamine-related cardiomyopathy who present in cardiogenic shock is challenging. We have highlighted the case of a young patient who was ineligible for advanced heart failure therapies and whose condition significantly improved by remaining abstinent and using continuous ambulatory inotropes as medical treatment. Our results suggest that ambulatory inotropes should be considered in the therapeutic arsenal when addressing challenging clinical pathologies, such as toxic cardiomyopathy.

**Novel Teaching Points**

• To identify the clinical presentation of amphetamine-related toxic cardiomyopathy.
• To consider alternative treatment in the management of cardiogenic shock attributed to amphetamine-related toxic cardiomyopathy.

## Supplementary Material

Supplemental Appendix S1

Video 1 Initial bedside echocardiography in parasternal long-axis view showing severe left ventricular hypokinesis and dilatation.

Video 2 Echocardiography in parasternal long-axis view performed after 30 months of abstinence and 18 months after weaning from inotropic support.
