# Supplementary material for: Exploring Ambulatory Continuous Inotropes as a Bridge to Recovery in Advanced Heart Failure Secondary to Amphetamine-Induced Cardiomyopathy
Source: CJC Open. 2024 Nov 16;7(1):23–6. doi: 10.1016/j.cjco.2024.10.013 (PMC11763879; doi:10.1016/j.cjco.2024.10.013)
Supplement: Supplemental Appendix S1 [file mmc1.pdf]

Supplemental Appendix S1

Ambulatory milrinone protocol infusion:

Potential candidates are evaluated for admissibility to the program during a heart failure hospitalisation. The conditions for admissibility to the program are the following :

- Patient Intermacs class 3 (inotrope dependent) or NYHA class 3-4 or stage 3D with optimal medical and pharmacological treatments or maximum tolerable doses
- Patient with an implantable automatic defibrillator with the exception of palliative care patients.
- Failure to wean milrinone during a hospital stay and documentation of deterioration of clinical condition in the absence of inotropes.
- Ability of the user to change the elastomeric infuser (photo included) with social support or a local clinic with nurse availability for assistance
- The risks and benefits of the therapy are accepted verbally by the user or his legal representative (hypotension, supraventricular and ventricular arrhythmias, torsade de pointes, headaches, syncope, catheter infection, bacteraemia, defibrillator shock, death).

The exclusion criteria are based on the clinical judgment of the treating physician and include the following :

- Intravenous milrinone not tolerated by the user during the hospital stay (refractory or uncontrolled ventricular or supraventricular arrhythmias, symptomatic hypotension)
- Cognitive problem compromising adherence to treatment
- Physical incapacity compromising adherence to treatment
- Means of communication (telephone) inaccessible
- User who does not have a refrigerator (conservation of medication)
- User who is homeless or living in an unsanitary environment
- Intravenous drug user
- Recent suicidal history

All patients are that benefit from home inotrope therapy are followed closely by our heart failure clinic with frequent in person and telephone consultations. Patients benefit from the up titration of goal directed medical therapy as well as frequent laboratory follow-up. There is also dedicated nursing availability for these patients in order to assist with troubleshooting in event of difficulties with the administration of medication.

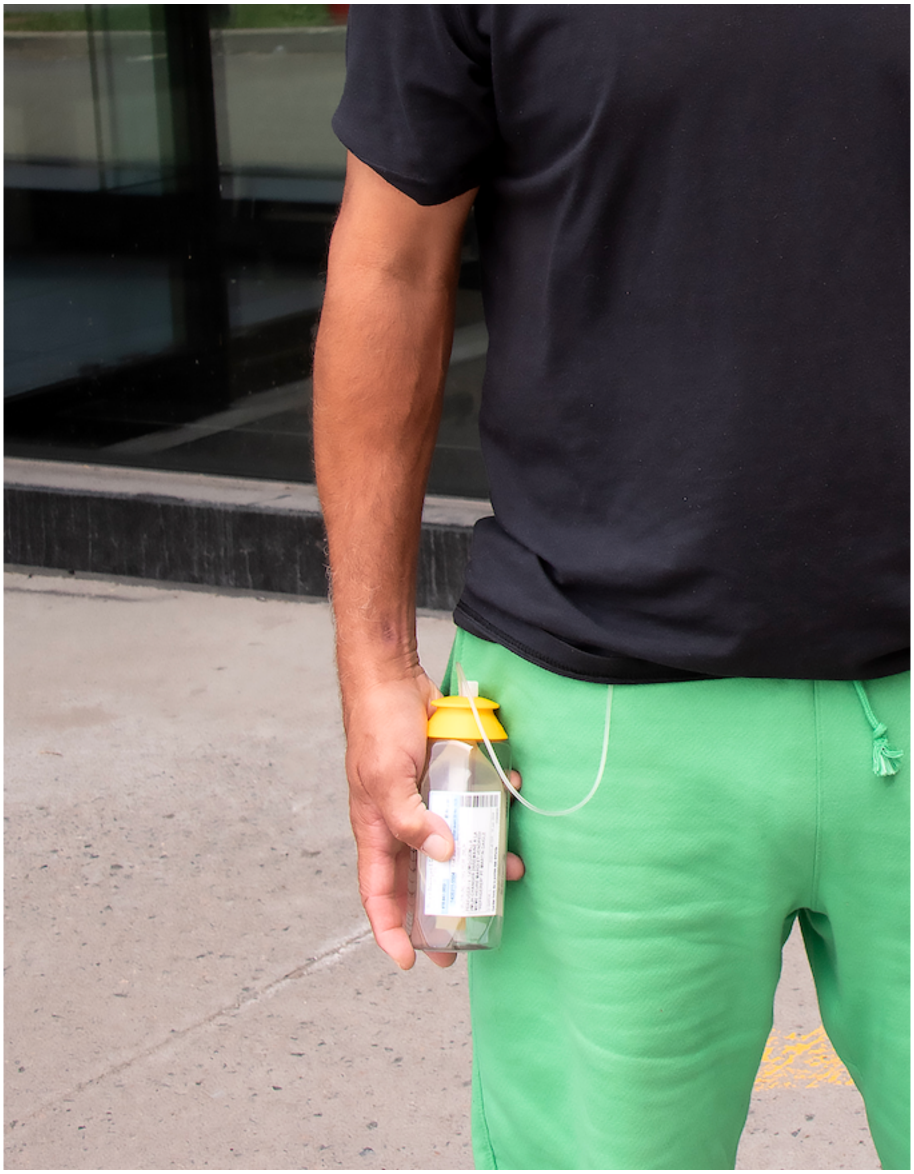

Supplemental Figure S1: Elastomeric infuser.

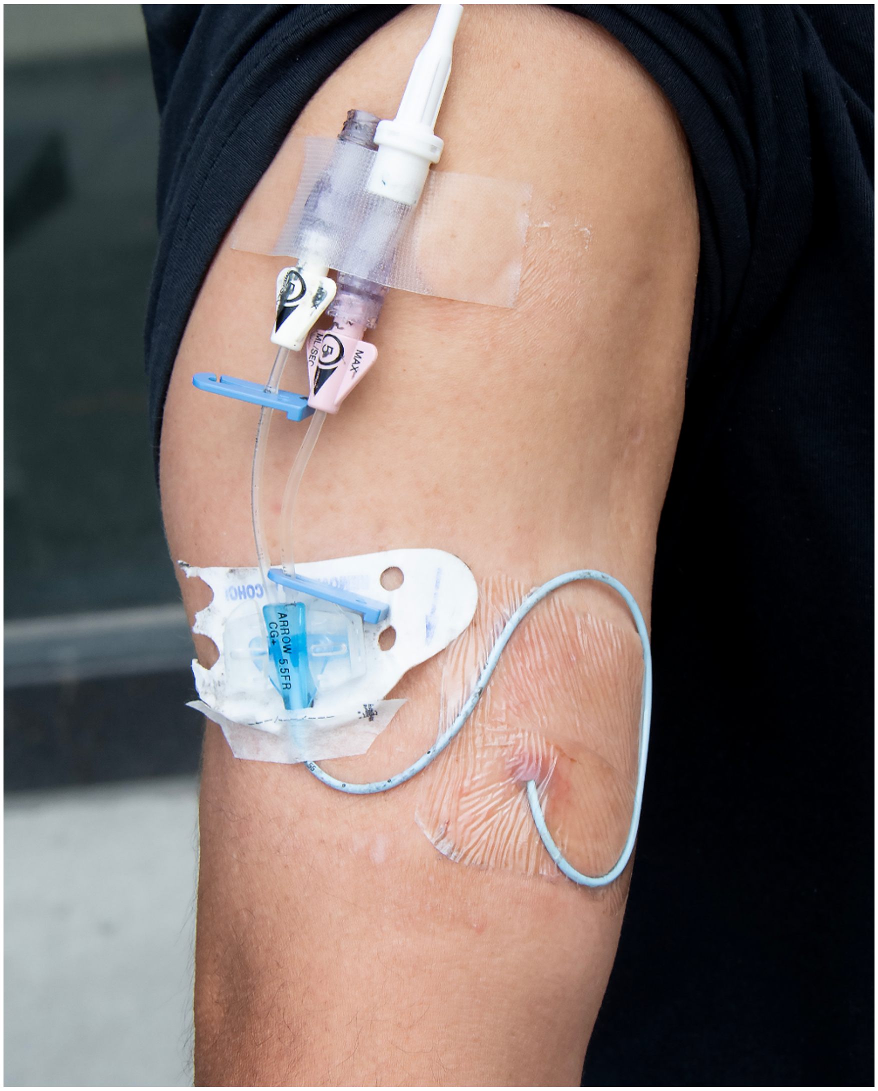

Supplemental Figure S2: Peripherally inserted central catheter (PICC) line.
